# The Interplay between Oxidative Stress and miRNAs in Obesity-Associated Hepatic and Vascular Complications

**DOI:** 10.3390/antiox9070607

**Published:** 2020-07-10

**Authors:** Jorge Infante-Menéndez, Andrea R. López-Pastor, Paula González-López, Almudena Gómez-Hernández, Oscar Escribano

**Affiliations:** Laboratory of Hepatic and Cardiovascular Diseases, Biochemistry and Molecular Biology Department, School of Pharmacy, Complutense University of Madrid, 28040 Madrid, Spain; jorgeinf@ucm.es (J.I.-M.); andreara@ucm.es (A.R.L.-P.); pgonza23@ucm.es (P.G.-L.)

**Keywords:** obesity, non-alcoholic fatty liver disease, atherosclerosis, cardiovascular diseases, oxidative stress, reactive oxygen species, mitochondrial dysfunction, NADPH oxidases, antioxidants, microRNAs

## Abstract

Nowadays, the obesity pandemic is one of the most relevant health issues worldwide. This condition is tightly related to comorbidities such as non-alcoholic fatty liver disease (NAFLD) and cardiovascular diseases (CVDs), namely atherosclerosis. Dysregulated lipid metabolism and inflammation link these three diseases, leading to a subsequent increase of oxidative stress (OS) causing severe cellular damage. On the other hand, microRNAs (miRNAs) are short, single-stranded, non-coding RNAs that act as post-transcriptional negative regulators of gene expression, thus being involved in the molecular mechanisms that promote the development of many pathologies including obesity and its comorbidities. The involvement of miRNAs in promoting or opposing OS in disease progression is becoming more evident. Some miRNAs, such as miR-200a and miR.421, seem to play important roles in OS control in NAFLD. On the other hand, miR-92a and miR-133, among others, are important in the development of atherosclerosis. Moreover, since both diseases are linked to obesity, they share common altered miRNAs, being miR-34a and miR-21 related to OS. This review summarizes the latest advances in the knowledge about the mechanisms of oxidative stress (OS) generation in obesity-associated NAFLD and atherosclerosis, as well as the role played by miRNAs in the regulation of such mechanisms.

## 1. Introduction

Obesity is currently considered a pandemic that results in an increased body weight owing to excessive fat accumulation. It is a chronic and multifactorial disease characterized by a pro-inflammatory environment in the adipose tissue. This situation might substantially provoke the development of adverse comorbidities linked to oxidative damage, such as type 2 diabetes mellitus, non-alcoholic fatty liver disease (NAFLD), cardiovascular diseases (CVDs) and cancer [1,2].

**Figure 1:**
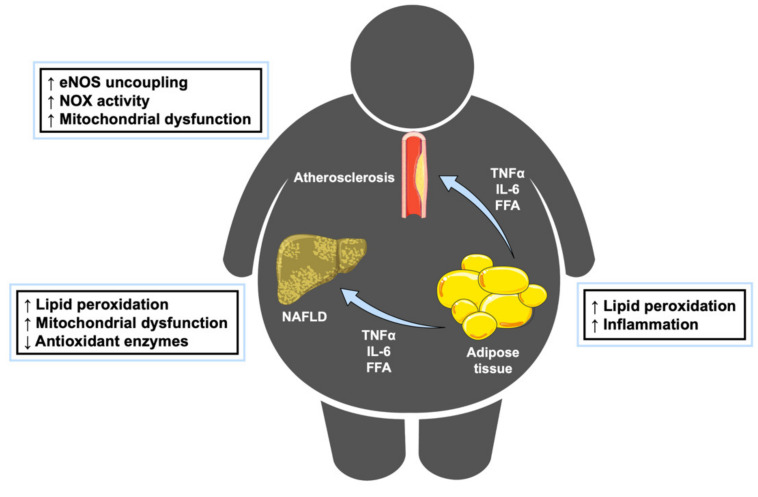
Obesity promotes the development of other conditions through inducing oxidative stress. Obesity is characterized by an oversupply of fatty acids, leading to lipid peroxidation and inflammation. Once adipose tissue capacity is surpassed, the release of FFAs and pro-inflammatory cytokines increases the risk of developing comorbidities, such as non-alcoholic fatty liver disease (NAFLD) or atherosclerosis. eNOS: endothelial nitric oxide synthase; FFA: free fatty acids; IL-6: interleukin 6; TNFα: tumor necrosis factor ɑ. This figure has been edited from Servier Medical Art. Servier is licensed under a Creative Commons Attribution 3.0 Unported License.

Oxidative stress (OS) arises from the imbalance between reactive oxygen species (ROS) production and detoxification. This state is involved not only in the progression of physiological processes such as aging, but also in the progression of several diseases, including obesity, atherosclerosis, non-alcoholic steatohepatitis (NASH) and cancer [3]. Particularly, in obesity, OS occurs due to an oversupply of fatty acids and glucose to mitochondria since obesity is often associated with hyperlipidemia and hyperglycemia [4]. ROS can be generated by multiple enzymes (e.g., oxidases, lipoxygenases, nitric oxide (NO) synthases, cytochrome P450 (CYP) isoforms) in different subcellular locations (such as the cytosol, the endoplasmic reticulum (ER), the mitochondrion or peroxisomes), and act as inflammation mediators [5]. Even though ROS are required for physiological processes, in large quantities, they are toxic to cells because they might damage macromolecules (DNA, lipids, proteins) [6,7]. The contribution of each mechanism differs depending on the disease despite having common sources of ROS, namely the mitochondrial electron transport chain (ETC) and NADPH oxidase (NOX) enzymes (Figure 1).

Mitochondria are the main source of ROS in the cell due to oxidative phosphorylation. ROS are formed in the ETC, where oxygen is the final electron acceptor, but also another source of ROS. Mitochondrial dysfunction plays a key role in pathological conditions such as NAFLD or CVDs, which will be observed in more detail throughout this review. This ROS burst fosters both mitochondrial DNA (mtDNA) and protein damage, as well as lipid peroxidation and membrane instability [8,9].

NOXs are enzymes belong to a 7-member family considered one of the main ROS sources in pathological conditions, such as cardiovascular and hepatic diseases [10,11]. In the liver, hepatocytes mainly express NOX1, NOX2 and NOX4 [12]. In the vascular system, rather than cell-specific NOX isoforms, each cell type expresses various members [13]. Owing to the important role that immune cells play in NAFLD and atherosclerosis, it is important to highlight the expression of NOX2 in phagocytes, like hepatic Kupffer cells [12,14,15]. These enzymes transfer electrons from NADPH to molecular oxygen, thus synthesizing superoxide.

In order to maintain the oxidative balance in the cell, different types of antioxidant mechanisms exist, enzymatic and non-enzymatic. Enzymatic antioxidants are located both at the mitochondria and the cytosol and include catalase (CAT), glutathione peroxidase (GPx), superoxide dismutase (SOD) or heme oxygenase-1 (HO-1) [5]. Regarding non-enzymatic antioxidants, two types are distinguished: endogenous (e.g., coenzyme Q, vitamin E) and exogenous (e.g., flavonoids, polyphenols).

Further development of new treatment or strategies for metabolic diseases is of great need. One of the novel tools for this purpose are microRNAs (miRNAs), a highly conserved class of short (20-22 nucleotides in length) and single-stranded non-coding RNAs. The majority of mammal gene expression is negatively regulated at post-transcriptional level by miRNAs [16,17]. Consequently, these molecules not only have a critical role in maintaining the oxidative balance in physiological processes, but also in promoting OS during the progression of pathological states including obesity, NAFLD and CVDs when altered. Despite the underlying mechanisms that these interesting molecules carry out are still poorly understood, manipulating the miRNA expression profile is required in order to be the driving force of the disease management worldwide.

In recent years, a big effort has been made to unravel the role that miRNAs play in the course of the development of pathologies. This review will provide an overview of the main molecular mechanisms that promote OS in NAFLD and CVDs, particularly atherosclerosis, as well as analyze the role of miRNAs in the regulation of OS in such conditions.

## 2. Oxidative Stress in NAFLD Progression

In Western countries, NAFLD is considered the most common hepatic disorder which affects more than 90% of obese, 70% of overweight people and around 25% of normal weight people [18]. This pathology represents the hepatic hallmark of metabolic syndrome [19]. Its development consists of the following stages: (1) an earliest stage of benign steatosis (fatty liver) and (2) NASH. Occasionally, the latter stage can lead to fibrosis and cirrhosis [20,21].

The progression mechanism from steatosis to NASH is unclear, although ROS generation and, as a result, OS has a leading role in this process [3,22,23,24]. In the liver, the increase in ROS formation, aside from the alteration in antioxidant systems, leads to lipid peroxidation followed by inflammation, stellate cell activation and, ultimately, fibrogenesis [22,23]. In addition to the reduction in hepatic antioxidant biomarkers [23,25], other OS biomarkers in NAFLD samples might be lipid damage byproducts (thiobarbituric acid reactive substances (TBARS), mainly malondialdehyde (MDA) and 4-hydroxynonenal (4-HNE)), DNA oxidation products like 8-hydroxy-2’-deoxyguanosine (8-OH-dG) and protein oxidation byproducts (carbonyl, nitrotyrosine, hydroxyproline) [25,26,27].

In NAFLD, hepatic steatosis induces an increase in the mitochondrial β-oxidation rate due to the higher supply of free fatty acids (FFA) to hepatocytes, raising the availability of electrons that flow through the ETC [6]. The inability of the ETC, due to the electron overload, to transfer these electrons to oxygen through complex IV allows them to react directly with it in other locations, thus forming ROS [9]. In order to alleviate the pro-oxidant pressure, hepatocytes activate the expression of uncoupling protein 2 (UCP-2), which dissipates the membrane potential across the mitochondrial inner membrane, allowing the cell to temporarily quench the increase in OS. This leads to long-term ATP depletion and energetic stress in the hepatocytes [24]. Furthermore, mitochondrial ROS impair the expression of peroxisome proliferator-activated receptor γ coactivator-1 ɑ (PGC-1ɑ) in fatty liver, a key regulator of mitochondrial biogenesis. Consequently, mitochondria in NASH patients are big and have crystalline inclusions, which indicate mitochondrial dysfunction and liver injury [22,28]. Mitochondrial dysfunction is also characterized by mitochondrial outer membrane permeabilization, promoting protein release to the cytosol, cell death and loss of mitochondrial integrity [29].

Hepatic lipid overload activates alternative lipid catabolism pathways such as microsomal ω-oxidation and peroxisomal β-oxidation. In NASH, the expression and activity of the microsomal CYP2E1 and 4A is increased. CYPs catalyze the oxidation of long chain fatty acids to long chain dicarboxylic acids, synthesizing superoxide in the process [30,31]. ω-oxidation products are subsequently sent to β-oxidation in peroxisomes, where acyl-CoA oxidases directly generate hydrogen peroxide (H_2_O_2_). Both ω- and β- oxidation are increased in NASH, leading to augmented generation of ROS and promoting OS. Moreover, peroxisomal β-oxidation products are further metabolized in the mitochondrial tricarboxylic acid cycle, thus the functionality of this organelle is exceeded [29,32].

A well-known source of ROS generation is through direct catalysis by iron, since it generates hydroxyl radical through the Fenton and Haber–Weiss reactions [24]. Iron can also affect the activity of antioxidant enzymes such as HO-1 and GPx. It is important to note that peroxisomes are rich in iron, which works as a cofactor in the reactions that take place in them, so iron leakage from abnormal peroxisomal function in NASH can further promote OS [33]. Other ROS generation mechanisms include NOXs. While NASH patients experience an increase in NOX4 expression [34], rodents with diet-induced steatosis present higher levels of NOX2 [35,36]. Even though these enzymes are altered in NAFLD, their contribution in the disease is unclear.

Lipid peroxidation is one of the main consequences of OS. In this process, ROS attack membrane lipids giving rise to 4-HNE and MDA. Such molecules are highly reactive and have a longer half-life than ROS, which allows them to rapidly diffuse and exacerbate oxidative damage [6]. Moreover, ROS can impair protein function through carbonylation and nitrosylation of amino acids, and by oxidizing thiol groups that are fundamental for iron-sulfur cluster function [9,27]. This elicits damage of mitochondrial components, inflammation and even fibrogenesis. Oxidative damage can also occur in the DNA, especially in the mitochondria [37], due to a lack of physical protection by histones or the nuclear membrane and absence of repair mechanisms. In turn, OS-driven mutations and deletions in the mitochondrial genome, where the ETC complexes are encoded, further reinforce ROS synthesis [29]. Oxidative byproducts, including those reactive aldehydes, can also react with proteins, inducing the formation of protein-protein and DNA-protein adducts and leading to the inhibition of the ETC complexes, exacerbating ROS production [24,25].

Regarding antioxidant protection in NAFLD, there are controversial findings in the literature. On the one hand, some studies have shown decreased expression of enzymatic antioxidants such as SOD1/2, CAT or GPx in the progression of NAFLD/NASH [25,27,38], while other authors reported unchanged or elevated expression and total antioxidant capacity [25,26,39]. This issue might be due to a need for antioxidant protection in the first stages of OS-driven NAFLD, but the lack of effectiveness results in a decrease in enzymatic antioxidant expression in advanced stages of NAFLD/NASH progression. 

### miRNA Modulation of Oxidative Damage in NAFLD

**Figure 2:**
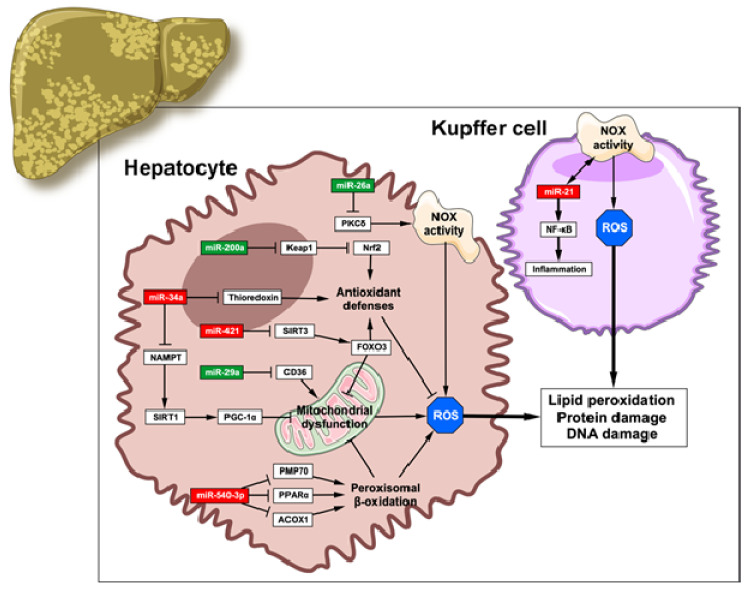
miRNAs as modulators of oxidative stress in NAFLD. Aberrant expression of miRNAs in hepatocytes (left) and Kupffer cells (right) interferes with the correct oxidative balance of the liver. miRNAs that promote *oxidative stress are highlighted in red, whereas those highlighted in green favor the maintenance of the balance. This figure has been edited from Servier Medical Art. Servier is licensed under a Creative Commons Attribution 3.0 Unported License.*

Over the last few years, owing to the urge to find a treatment for NAFLD, the knowledge regarding miRNAs that regulate OS in this disease has rapidly increased. Indeed, these molecules have been described to promote OS in NAFLD (Figure 2).

As mentioned, mitochondrial dysfunction is a pivotal mechanism of OS generation in NAFLD due to the oversupply of electrons to the ETC. Several miRNAs favor this malfunctioning, while others play a protective role. A high-fat diet (HFD)-induced NAFLD model showed increased levels of miR-421, which results in downregulation of sirtuin 3 (SIRT3), a forkhead box O3 (FOXO3) activator. FOXO3 grants protection against OS by inducing CAT and SOD2 expression; thus, miR-421 promotes oxidative damage by inducing mitochondrial dysfunction [40]. Similarly, liver-specific augmenter of liver regeneration (ALR) knock-out mice submitted to a HFD along with fructose and sucrose drinking water showed an altered miRNA expression profile, exhibiting marked overexpression of the novel miR-540-3p. This miRNA functions as a negative regulator of peroxisomal lipid oxidation by targeting peroxisome proliferator activated receptor ɑ (*Ppara*), acyl-CoA oxidase 1 (*Acox1*) and peroxisomal membrane protein-1 (70 kD) (*Pmp70*), ultimately promoting mitochondrial dysfunction [41]. In contrast, the re-expression of miR-29a has recently been described as a potential protector mechanism in NAFLD progression, since its levels are usually decreased in this disease. Lin et al. observed that an important miR-29a target is cluster of differentiation 36 (CD36), a fatty acid importer that promotes mitochondrial damage by hyperactivating β-oxidation and the production of ROS; therefore, increasing the levels of miR-29a could avoid the CD36-mediated deleterious effects [42].

ROS generation by NOXs, which play a huge role in OS, is also regulated by some miRNAs. More precisely, miR-26a levels decrease in HFD-fed mouse models [43]. In an in vitro model of hepatosteatosis, miR-26a downregulated protein kinase C δ (PKCδ), a kinase that promotes ROS generation by activating some NOX subunits [44], induced a reduction in MDA levels and increased antioxidant capacity. Therefore, miR-26a could protect against steatosis progression to NASH [45]. Intriguingly, miR-21 is an OS-responsive miRNA whose overexpression depends on NOX2 activation [46,47]. As a result, nuclear factor κB (NF-κB) is activated, aggravating liver inflammation by inducing a shift in Kupffer cells to a pro-inflammatory phenotype [46]. In addition, pro-fibrogenic effects were attributed to miR-21 due to its regulation on SMAD member 7 (SMAD7), which inhibits the activity of transforming growth factor ꞵ (TGF-ꞵ) [47].

Lastly, miRNAs also control the levels of antioxidant enzymes. For instance, miR-34a activity promotes OS development in NAFLD both directly and indirectly. By targeting thioredoxin, a well-known antioxidant, miR-34a overexpression aggravates oxidative damage [48]. Others reported a decrease in NAD^+^ levels due to miR-34a targeting nicotinamide phosphoribosyltransferase (NAMPT), which affects sirtuin 1 (SIRT1) levels and activity. Reduction of this deacetylase supposes a negative regulation of genes involved in lipid metabolism, such as PGC-1ɑ and sterol regulatory element binding protein 1c (SREBP-1c), suppressing lipid oxidation and fostering lipid synthesis and inflammation [49]. On the contrary, Kelch-like ECH-associated protein 1 (Keap1), a negative regulator of nuclear factor erythroid 2-related factor 2 (Nrf2), is targeted by miR-200a. Fructose administration induced steatosis and liver injury and repressed miR-200a expression in rats. This led to exacerbated inhibition of Nrf2 and its target genes (glutathione S-transferase, HO-1…), aggravating OS damage in NAFLD [50].

## 3. Pro-Atherosclerotic Oxidative Stress Mechanisms

The main cause of death in Western countries are CVDs, which appear in most cases as a result of atherosclerotic processes [51,52]. Atherosclerosis is defined as an inflammatory disease in which lipids are accumulated in the artery wall, resulting in its thickening, vascular lumen narrowing and prompting ischemia. The atherosclerotic process is characterized by a series of events: (1) endothelial dysfunction (ED) appears, (2) attracted macrophages differentiate into foam cells, (3) vascular smooth muscle cells (VSMCs) migrate and proliferate causing intimal hyperplasia, (4) eventually, angiogenesis, VSMC apoptosis, fibrosis and plaque rupture occur [53].

The endothelium is the main regulator of vascular function, since NO synthesis in endothelial cells (ECs) mediates VSMC relaxation and vasodilation. Endothelial activation is induced by pro-inflammatory cytokines, increasing the expression of adhesion molecules in the surface of ECs to allow immune cell adhesion and extravasation. Sustained activation of the endothelium in atherosclerosis is driven by an imbalance between vasodilator and vasoconstrictor agents, with pro-inflammatory and pro-thrombotic character, reducing NO bioavailability and, ultimately, promoting ED [54]. 

The onset and progression of atherosclerosis is led by inflammation-driven OS, which in turn induces more inflammation [55]. The main OS-promoting mechanisms that induce vascular damage include endothelial nitric oxide synthase (eNOS) uncoupling, superoxide hyperproduction by NOXs and mitochondrial dysfunction. 

OS is induced by eNOS uncoupling, promoting ED. eNOS activity requires the participation of many cofactors, including tetrahydrobiopterin (BH_4_); therefore, BH_4_ availability is key for eNOS malfunctioning, as superoxide synthesis occurs in this situation instead [56]. In this scenario, higher superoxide production reacts with NO yielding peroxynitrite, which reduces NO bioavailability and induces protein nitration [57,58]. In addition, inducible nitric oxide synthase activation (iNOS) seems to be involved in eNOS uncoupling, since its activity occurs as a burst that promotes cofactor depletion in response to pro-inflammatory signals [24,59].

ROS signaling is fundamental for the maintenance of vascular function, since under physiological conditions NOX2 and NOX4 expression prompt both EC proliferation and survival. However, NOX isoforms also function as a direct source of ROS in atherosclerosis [60]. NOX2 activity is higher in the endothelium and macrophages of *ApoE^-^*^/-^ models of atherosclerosis prior to the occurrence of atherosclerotic lesions, demonstrating the role of OS in early stages of atherogenesis [61]. Furthermore, several assays carried out on *ApoE^-^*^/-^ mice with *Nox1* or *Nox2* deletion and fed with a HFD showed an improvement in aortic superoxide and NO levels, lower inflammation and decreased plaque area [61,62]. Lastly, NOXs might also be involved in eNOS uncoupling by inducing BH_4_ oxidation [56]. The role of NOX4 in atherosclerosis is still not clear. On one hand, its persistent activation is supposed to be detrimental, mediating CVD in aged and hyperlipidemic mice. In this regard, the overexpression of NOX4 in artery wall of aged subjects aggravates atherosclerosis [63,64]. However, a possible protective role of NOX4 in atherosclerosis has been described since this isoform produces low levels of H_2_O_2_, reducing the inflammation status and, consequently, atherosclerosis severity [65]. 

Physiologically, as it has been mentioned, ROS are generated by mitochondrial complexes. However, in atherosclerosis, a role has been described for the p66 isoform of SHC adaptor protein 1 (p66^Shc^), a mitochondrial protein involved in cytochrome c oxidation and subsequent ROS formation [66]. In this sense, cell death signals arise. Moreover, taking into consideration that cytosolic ROS can diffuse to other cellular compartments, NOX-derived oxidative damage can also contribute to mitochondrial injury. Indeed, the atherosclerotic lesion is characterized by a decreased respiration rate due to oxidative mtDNA damage, thus decreasing the number of copies and the expression of the ETC complexes I, II and IV [8]. On the other hand, eNOS uncoupling-induced peroxynitrite formation might cause mitochondrial damage through membrane lipid oxidation and ETC complex damage, as well as SOD2 [58,67].

OS is responsible for early events in atherogenesis. As a consequence, lipid peroxidation, protein carbonylation and mtDNA damage are known to occur in this process [27], contributing to plaque vulnerability. Vascular oxidative damage results in low-density lipoprotein oxidation (oxLDL). Persistent exposure of vascular cells to oxLDLs and binding to their receptor, lectin-type oxidized LDL receptor 1 (LOX-1), exacerbates ROS production and inflammation [68]. The involvement of these molecules is important in the different stages of the atherosclerotic process since oxLDL-mediated ROS production is enhanced through NOX induction [69]. In addition, the increasing ROS production damages DNA, protein and lipids by the previously mentioned mechanisms in NAFLD. 

### miRNAs Linking Atherosclerosis and Oxidative Stress

**Figure 3:**
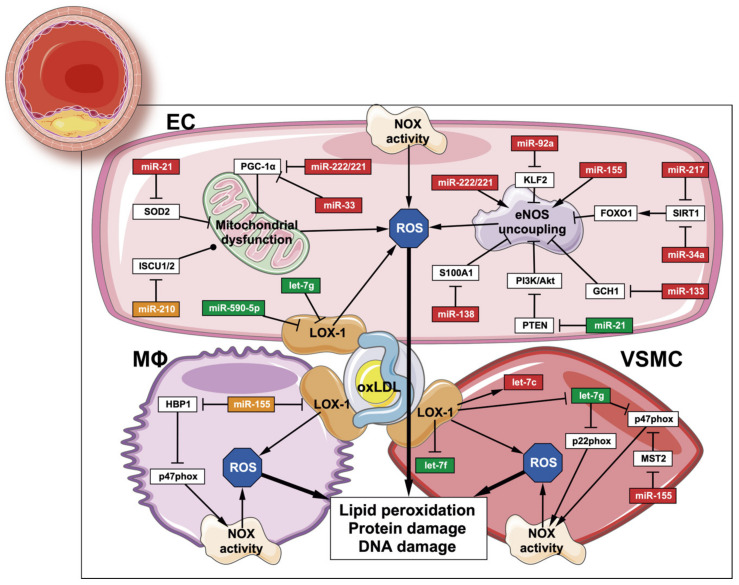
miRNAs regulate oxidative damage in atherosclerosis. Dysregulation of oxidative stress-promoting mechanisms is driven by miRNAs in endothelial cells (upper half), macrophages (lower half, left) and vascular smooth muscle cells (lower half, right). Oxidative stress is promoted by miRNAs that are highlighted in red, whereas the normal oxidative balance is maintained by the ones highlighted in green. For those highlighted in orange, both roles have been described. EC: endothelial cell; Mφ: macrophage; VSMC: vascular smooth muscle cell. This figure has been edited from Servier Medical Art. Servier is licensed under a Creative Commons Attribution 3.0 Unported License.

Recently, elucidating the involvement of OS in atherosclerosis and its relationship with miRNAs has awakened great interest. As a result, reducing miRNA-mediated OS and upregulating miRNAs with antioxidant properties are long-term goals for atherosclerosis treatment (Figure 3).

Given that eNOS activity is crucial for the correct vascular function, miRNAs regulating its function can either promote or protect against ED. The presence of oxLDLs induces an overexpression of miR-92a, targeting Krüppel-like factor 2 (KLF2). Subsequently, this regulation negatively affects eNOS, promoting ED in atherosclerosis [70,71]. Another predicted target of this miRNA are NOX proteins, which might exacerbate oxidative damage [72]. Other miRNAs such as miR-34a and miR-217 are upregulated in atherosclerotic lesions, both of which are able to target SIRT1 [73,74]. SIRT1 deacetylates forkhead box O1 (FOXO1), promoting eNOS transcription and activity [75,76]. Therefore, the upregulation of SIRT1-regulating miRNAs could suppose a new pathogenic mechanism in atherosclerosis regarding eNOS activity. Regarding eNOS expression, miR-222/221 also exert a negative regulatory action on this enzyme [77,78]. Nevertheless, since these miRNAs do not show binding sites in nitric oxide synthase 3 (*NOS3*) mRNA, the exact regulatory mechanism is yet to be unraveled [77]. Aside, miR-138 targets S100 calcium-binding protein A1 (S100A1), a protein that plays a critical role in eNOS activity. Hypoxia-induced miR-138 expression decreases the levels of S100A1, resulting in the loss of eNOS activity [79]. EC dysfunction might be partially restored by lovastatin treatment through blunting miR-133 overexpression. This miRNA is upregulated by cytokines and pro-oxidant mediators and targets GTP cyclohydrolase 1 (GCH1), an enzyme involved in BH_4_ synthesis and, consequently, in eNOS uncoupling [80].

Regulation of NOX activity by miRNAs is key for the maintenance of the oxidative balance in the atherosclerotic environment. Dysregulation of let-7 family is commonly found in CVDs, since some of its members (let-7g, let-7f) are downregulated in atherosclerotic plaques [72], VSMCs treated with oxLDLs [81] and others (let-7c) are upregulated [82]. Moreover, let-7g is able to target p22^phox^ and p47^phox^ and downregulate their mRNA levels, both of which belong to the NOX complex, suggesting a protective role of this miRNA against oxidative damage [81]. Reduced ROS formation in ECs is promoted by let-7g and miR-590-5p, whose target is LOX-1. However, miR-590-5p expression is downregulated by angiotensin II (Ang II), and taking into account that Ang II levels are higher in obesity, this regulation could suppose a source of ROS [81,83,84].

The pro-atherogenic effect of miR-221/222 and miR-33 seems to be critical, altering mitochondrial biogenesis and OS. Their overexpression leads to a decrease in PGC-1ɑ, ultimately inducing mitochondrial dysfunction in the endothelium [85,86]. In addition, miR-33 knock-out also decreases the expression of pro-oxidant enzymes, e.g., iNOS [87]. Considering iron metabolism, in response to hypoxia, the inhibition of iron-sulfur cluster assembly enzymes 1/2 (ISCU1/2) is mediated by miR-210. As a result, the assembly of FeS clusters in ETC is impaired, decreasing the production of ROS. However, in conditions of normoxia, miR-210 promotes ROS flux in the mitochondria by targeting ISCU [88,89].

Some other miRNAs, such as miR-21 or miR-155, are master regulators of OS in the atherosclerotic process owing to their participation in many simultaneous mechanisms. In the literature, the role of miR-21 in response to oxidative damage is still unclear. For instance, Weber et al. demonstrated that the beneficial increase in eNOS activity and NO production is due to miR-21 targeting phosphatase and tensin homolog (PTEN), thus restoring the phosphatidylinositol 3-kinase (PI3K)/Akt/eNOS pathway and protecting against OS-stimulated apoptosis [90]. Additionally, Lin et al. studied the involvement of miRNAs in H_2_O_2_-mediated gene regulation in VSMCs, showing the upregulation of miR-21. More precisely, this miRNA might be essential in the suppression of ROS-induced VSMC death, since another of its targets is programmed cell death-4 (PDCD-4) [91]. On the other hand, eNOS inhibition by asymmetric dimethyl arginine (ADMA) in angiogenic progenitor cells in vitro and in coronary artery disease patients, which reduces NO bioavailability, resulted in higher miR-21 levels and a consequent indirect SOD2 downregulation. This reinforces the idea of the central role of NO signaling in maintaining the vascular redox balance [92].

In the case of miR-155, the treatment with simvastatin has an anti-atherosclerotic effect, as it attenuates tumor necrosis factor ɑ (TNFɑ)-induced miR-155 expression and derepresses eNOS activity [93]. The endothelial overexpression of miR-155 also promotes a decrease in OS by targeting BTB and CNC homology 1 (BACH1), which ultimately increases HO-1 expression [94]. Silencing of miR-155 might be responsible for an increased oxLDL-stimulated lipid uptake, LOX-1 upregulation and increased release of pro-inflammatory cytokines (interleukin (IL)-6, IL-8 and TNFɑ). Aside from LOX-1, other scavenger receptors, such as CD36 and cluster of differentiation 68 (CD68), are regulated by this miRNA [95]. In this sense, oxLDLs promote miR-155 overexpression in macrophages, which targets HMG box transcription factor 1 (HBP1), a p47^phox^ inhibitor, giving rise to the production of ROS and differentiation to foam cells [96]. Furthermore, miR-155 overexpression in VSMCs promotes cell proliferation, an increase in ROS formation and, consequently, in OS. Specifically, through targeting of the extracellular signal-regulated kinase (ERK) pathway inhibitor serine/threonine kinase 3 (MST2), p47phox and NF-κB expression are promoted by this miRNA [97].

## 4. Diet-Derived Compounds as Antioxidant Defense through miRNAs Modulation

It is already well known that maintaining a healthy lifestyle by a combination of a varied diet and exercise reduces the risk of developing diseases such as obesity, CVDs and NAFLD. Recently, the effect of the diet on OS has begun to be highlighted due to its relationship with obesity and its comorbidities [98].

Feeding with HFD increases body weight, promotes lipid accumulation and induces the establishment of a pro-oxidant state [27]. This evidence highlights the importance of maintaining a well-balanced diet rich in nutrients with antioxidant properties, which are most commonly found in edible plants. The need for the implementation of such modifications is rising along with the extent of the obesity pandemic, mainly due to a higher intake of processed foods rich in saturated fats and sugar and sedentary lifestyle.

Recently, more studies are focusing on the effects that the introduction of antioxidant compounds can have in the expression of miRNAs. For instance, resveratrol can exert an anti-inflammatory effect through the modulation of inflammation-related miRNAs: patients with hypertension under a diet supplemented with resveratrol showed higher expression of miR-21, as well as a reduction in the levels of miR-155 and miR-34a [99].

Many compounds with antioxidant properties regulate miR-34a. Pterostilbene, a resveratrol analogue found in edible berries, downregulates miR-34a overexpression in fructose fed rats, leading to the restoration of SIRT1 activity and the inhibition of SREBP-1 lipogenic activity [100]. Melatonin is naturally found in edible plants and may be responsible for lower expression of miR-34a in melatonin-treated HFD-fed mice, along with higher SIRT1 and lower SREBP-1 activities [101]. Finally, the novel carnosic acid decreases miR-34a overexpression, alleviates dyslipidemia and activates the anti-apoptotic properties of SIRT1 in rats submitted to a HFD [102].

Moreover, curcumin also has beneficial properties, since this compound restores the expression levels miR-200a in fructose fed rats, blocking the activation of the inflammasome [103]. miR-200a is also induced by polydatin, a plant polyphenol, leading to a stronger regulation of miR-200a on Keap1 and antioxidant defense activation by Nrf2 [50]. In consequence, introducing the previously mentioned compounds in the diet of the population might have beneficial effects against NAFLD and CVDs.

## 5. Discussion

The prevalence of obesity has undergone a great rise worldwide in recent years. Together with this health threat, its comorbidities will continue increasing over the following years [1,2]. Adipocytes secrete adipose-specific proteins, adipokines, that impact other tissues maintaining their metabolic function; however, in obesity, this secretory phenotype switches from beneficial adipokines to pro-inflammatory cytokines, leading to whole-body inflammation [104]. Due to the chronic, systemic, low-grade inflammation state that obesity is related to, obese individuals develop a plethora of metabolic alterations in different organs that are grouped under the term ‘metabolic syndrome’, which includes NAFLD and atherosclerosis [3,105]. Simultaneously, during the development of obesity, the adipose tissue experiments an increase in ROS production and OS, in part due to the FFA accumulation in the adipocytes and the switch in the adipokine secretion profile [3]. Consequently, oxidative damage in obesity contributes to OS in peripheral tissues, accelerating the development of obesity-associated metabolic disorders [105].

As the hazard of obesity and its related conditions increases, there has been a need to acquire a healthier lifestyle by introducing a balanced diet and exercise which improve the severity of OS [59,106,107]. Indeed, over the last few years, the benefits of many natural and artificial compounds on oxidative damage have been tested. In this regard, polyphenols are the most studied antioxidants. For instance, the polyphenolic SIRT1 activator resveratrol improves mitochondrial dysfunction and antioxidant capacity in both NAFLD and CVDs [108,109]. Furthermore, the transcriptional activity of Nrf2 is induced by apigenin, a flavonoid polyphenol, and by plant polysaccharides, promoting antioxidant defenses in NAFLD [110,111,112]. Nut polyphenols have also shown protective effects against CVDs, reducing lipid peroxidation and increasing antioxidant levels [113,114,115]. Many trials have used vitamin E to quench OS in NAFLD, and although beneficial effects have been observed showing an improvement in hepatic injury markers like alanine aminotransferase, the exact protective mechanism remains unknown [116]. Even though the cardioprotective effect of antioxidant compounds has not been deeply studied, Li et al. have described that ursolic acid, a terpenoid, has anti-atherosclerotic properties in ApoE^-/-^ mice by inhibiting ROS-induced LOX-1 expression [117].

miRNAs are important gene expression regulators that promote physiological transitions and, therefore, could be involved in the development of diseases. Since these molecules can be secreted by cells in extracellular vesicles, not only are they able to finely regulate gene expression in the tissues in which they are expressed, but also in distant cells when these vesicles are secreted to the bloodstream [118]. In this sense, extracellular miRNAs may represent a means of communication between cells that could promote the progression to a pathological state [119]. miRNAs regulate broad sets of genes, including those that are involved in OS control. Consequently, it is of great interest to unravel the role that specific miRNAs play in controlling specific enzymes and transcription factors related to OS.

Much effort has been made to determine the miRNA expression signatures in obesity, NAFLD and atherosclerosis. Although some common miRNAs are altered in these diseases, such as miR-21 or miR-34a, their up or downregulation depends on the specific condition. Conversely, other miRNAs expression is specifically disturbed in each disease; for instance, the targeting ability of miR-421 on SIRT3 and miR-200a on Keap1/Nrf2 suggests that normalizing their expression might grant protection against OS-induced damage in NAFLD. Likewise, restoring the miR-92a/NOX and miR-133/GCH1 regulatory axes could ameliorate oxidative injury in atherosclerosis. Some miRNA-based therapies have already been proposed for the treatment of obesity comorbidities, such as antisense miR-122 therapy for NAFLD [120] and antisense miR-33 for atherosclerosis [121,122], although none of them specifically intend to reduce OS. For this reason, taking into account the prominent role that OS plays in obesity and its comorbidities, discovering dysregulated miRNAs that regulate ROS generation in this context could suppose a breakthrough in their treatment.

## 6. Conclusions

OS is a key mechanism involved in the development of both NAFLD and CVDs. Different types of antioxidant compounds have been studied to protect against these diseases; however, taking into account the ability of miRNAs to target specific proteins, they have been proposed as possible novel treatments to alleviate OS in obesity-related comorbidities. However, there is a need for deepening the knowledge of the perils that OS-promoting mechanisms have in these diseases, and the potential that miRNAs may have as therapeutic targets.
